# The role of diverse leadership styles in teaching to sustain academic excellence at secondary level

**DOI:** 10.3389/fpsyg.2022.1096151

**Published:** 2023-01-09

**Authors:** Samra Maqbool, Hafiz Muhammad Ihsan Zafeer, Pingfei Zeng, Tamara Mohammad, Osama Khassawneh, Ling Wu

**Affiliations:** ^1^College of Teacher Education, College of Education and Human Development, Zhejiang Normal University, Jinhua, China; ^2^School of Education, Shaanxi Normal University, Xi’an, China; ^3^College of Business Administration, American University in the Emirates, Dubai, United Arab Emirates; ^4^Lazaridis School of Business and Economics, Wilfrid Laurier University, Waterloo, ON, Canada

**Keywords:** strategic leadership, culture leadership, instructional leadership, sustaining academic excellence, secondary schools

## Abstract

Educational leadership is a multifaceted area of study. Unquestionably, leadership is the most deliberate field within the social sciences. Still, administrators have evaded the notions of leadership concept like a haunting tune. This study has focused particularly on the significance of varied leadership styles in teaching to sustain academic excellence at the secondary school level. The quantitative research method was used. Data was collected through the scale of diverse leadership styles (strategic, cultural, instructional leaderships and sustaining academic excellence) from 103 secondary schools in Punjab, Pakistan. The sample consisted of 540 teachers who were enacting as teachers presently. Based on research objectives and questions, two hypotheses were formed and tested using mean analysis to determine the average ranking of leadership styles. Pearson correlation to know the statistically significant relationship between each leadership styles, and overall scales with sustaining academic excellence. The results revealed that most teachers give preference strategic Leadership, then instructional leadership, and finally cultural leadership in their teaching to sustain academic excellence. Moreover, the findings also indicated that a statistically strong positive relationship among diverse leadership styles in teaching and sustaining academic excellence with the value (*r* = 0.752). Based on the findings, it has been concluded that when teachers increase their effort in the use of strategic, instructional, and cultural leadership styles, academic excellence may also sustain and improve.

## Introduction

Diverse leadership styles show an invaluable role in the victory and development of schools. Keeping up with changing times requires a renewal of educational movements. Its goal is to integrate education into a sustainable development agenda 2030 (SD) headed by the United Nations Organization (UNO) has outlined advancements in education regarding societies and futures based on sustainability in the twenty-first century. Developing a wonderful culture within a school largely depends upon the school’s leadership. In addition to this, a positive impact can be seen in a student’s learning and performance.

As indicated by Okilwa and Barnett (2017) in the 21st century, school leadership is essential for re-imagining vision, the mission, and goals of the schools (Naidoo and Petersen, 2015). Leadership Styles play a vital contribution to schools’ success and progress. Keeping up with changing times requires a renewal of educational movements (Ucar and Dalgic, 2021). However, the three leadership styles are prominent in school settings such as strategic, cultural and instructional leadership (Murphy et al., 2006).

Moreover, *strategic leadership* entails directing the effort of continual school improvement (Davies, 2003). While *culturally responsive leadership* promotes inclusive educational environments for ethnically and culturally diverse students and families. Culture is also based on shared beliefs, ideologies, values, assumptions, expectations, attitudes, and norms centered on learning (Madhlangobe and Gordon, 2012). Most of the time, *instructional leadership* is associated with school administrators, who are responsible for the academic success of every student enrolled in their schools and supervised curriculums, finances, and timetables (Southworth, 2002).

In general, leadership is within the framework of examining the extra subtle effects of unique management on various significant student outcomes (Sun et al., 2017). Educational leaders are problem solvers and facilitators, and a superior education must be offered through excellent teaching facilities. In schools that formalize teaching and learning, the role of the teacher is critical for educating society’s youth (Williams-Boyd, 2002).

Further, the instructional unit imposes excessive requirements for the skilled application of practice and evaluation. It creates protocols and systems for assessing how well preparation produces results in an environment conducive to all students’ education. Leaders must develop collaborative structures in schools to ensure students have distinctly enticing educational experiences (Hallinger, 2010).

In Pakistan, a school’s leadership role is non-negotiable and is often referenced in terms of its development since the education sector creates accountable inhabitants and human power to facilitate the achievement of the National Education Goals (NEGs) (Ali and Tahir, 2009). Achieving the state’s educational objectives (Bank, 2002). Many private schools in Pakistan contribute to achieving the Millennium Development Goals (MDGs). A fundamental right, education has become a national agenda, incorporated in the Sustainable Development Goals (SDGs), which encompass the Millennium Development Goals and the “Education for all” initiative (Dhindsa, 2016). Schools, colleges, and universities are all part of a national goal. The secondary sector is the most significant and fundamental sub-sector of education. A mattress rock supports the entire pyramid of education. According to research conducted in Pakistan and other developing nations, major training has the highest social and private costs of return compared to secondary and tertiary training.

Additionally, school is generally most successful during the first few years; subsequent years are generally less productive. Based on the academic and socio-economic indicators of developed and developing countries, it is evident that regular training and, most significantly, education and literacy have profound, direct, and identifying implications for the overall development of a nation. Countries in this area, such as China, South Korea, Malaysia and Singapore which have experienced remarkable growth in essential training, have also been able to attain high *per capita* incomes and sustain them (GoP, 2017). Factors that contribute to academic quality in teaching and learning include; (a) lecturer engagement, (b) presentation organization, (c) material that is tailored to assist students in meeting course learning goals, and (d) the compatibility of the lecturer’s teaching style with the subject matter. In light of this background, the researcher examines the nature of teaching and learning and the role of strategic leadership and management in developing and sustaining academic excellence.

Thus, diverse leadership styles in which strategic leadership style in teaching is the use of strategy to classroom management. Teachers employ this approach to engage students, maintain good morale, and fulfill the institution’s strategic objective of academic excellence and achievement. Educators may evaluate the strengths and limitations of their pupils and design successful engagement strategies with the aid of cultural leadership styles. Since the approach is inclusive, it works better for talented but disadvantaged children and enables instructors to fulfill students’ particular needs more effectively. Many educators and administrators use an instructional leadership style because it stresses concurrently enhancing teaching performance and student growth.

However, it is of the utmost importance that teachers develop leadership capabilities, since this is a vital need for improving the instructional quality of teachers both within and outside the classroom. Teacher leaders have superior class management abilities and are better able to motivate pupils to achieve academic achievement. In the present corpus of research, few studies have examined the support of diverse leadership styles in teaching on student learning and academic performance/excellence (Ucar and Dalgic, 2021). The core objective of this research is to explore the contribution of teaching in generating academic excellence using strategic leadership, Culture Leadership, and Instructional Leadership. And to know the association leadership styles in sustaining academic excellence. To fulfill the study’s objectives two research questions were made.

### Research questions

1. What is the role of strategic, cultural and instructional leaderships in teaching to sustain academic excellence at the secondary level?
2. Is there any relationship of sustaining academic excellence between strategic, cultural and instructional leadership in teaching at the secondary level?

### Hypothesis

1. There is a significant role of strategic, culture and instructional leaderships in teaching to sustain academic excellence at the secondary level.
2. There is a statistically significant relationship between strategic, culture and instructional leaderships in teaching to sustain academic excellence at the secondary level.

### Review of literature

In a recent discussion, the question of whether academic excellence is an inborn quality was raised (Gosling and Hannan, 2007). Techniques and skills were at issue (Kane et al., 2004). Some studies rated teachers highly for their personality attributes, such as approachability, passion, and enthusiasm (Enakrire, 2021). The importance of leadership skills and expertise increased for students as they progressed through higher levels of academia (Yielder and Codling, 2004).

Tschannen-Moran and Gareis (2004) effective schools are built on the foundation of perfect leaders. As far as the teacher is concerned, the major and senior administrative groups play a crucial role in initiating employment by raising the expectations of both students and teachers. The school transformation movement seeks to enhance student achievement, and as a result, calls for innovative management (Eacott, 2013). Keeping ‘quality leaders’ and filling management positions with outstanding individuals is a challenge faced by educational administrators and schools (Bin Mohd Ali and Ali, 2015).

The theory of organizational change is examined as a result of the establishment of communities of practice (Hussain et al., 2018). It relates to ongoing changes in school environments and the role of instructional leaders in facilitating these changes. Finally, the framework emphasizes continuous improvement within the complex organization of schools (Adolfsson and Alvunger, 2017).

Furthermore, contingency leadership theory, also known as adaptive leadership. This theory is based on the context in which a leader operates. This concept states that situational consequences determine success or failure. The situational context directly impacts the effectiveness of a leader (Anderson and Sun, 2017). While the personality of a leader is important, context and situation have a greater impact on success. The theory holds that competent leaders can adjust their leadership styles in response to the situation’s requirements. However, an example of the contingency concept is Hershey and Blanchard’s Situational Theory, Evans and House’s Path-Goal Theory, and Fiedler’s Contingency Theory (Masood et al., 2006; Amanchukwu et al., 2015). An institution’s culture is a set of values and beliefs that form the institution’s identity and are fundamental to improving educational institutions (Krüger et al., 2007). As part of the process of establishing a school’s culture, the school’s long-term goals and direction are set, and traditions, rules of behavior, values, and school visions are used to give the school a sense of cohesion and unity (Tzianakopoulou and Manesis, 2018). School culture is also responsible for ensuring a stable environment within the school community, which contributes to students’ academic excellence (Turan and Bektas, 2013).

However, in school settings leadership is the ability to plan, control, lead, organize, and manage human and material resources to accomplish school goals (Saleem et al., 2020). Global education policy programs emphasize leadership style. It influences teacher motivations, skills, school atmosphere, and environment to improve educational results. A growing body of evidence from research and experience demonstrates that the major responsibility of educational leaders is to place a focus on student accomplishment by creating demanding, caring, and supportive learning environments.

### Relationship between leadership styles and academic excellence

In primary schools, Williams (2009) discovered a favorable correlation between leadership style and school culture. In Iowa primary schools, Decker (1989) found no correlation between leadership style and school environment. In addition, Kennedy (2021) found no correlation between leadership style and school climate in New Jersey. Similarly, Nichols (2016) finds comparable outcomes in an urban school system. There was a strong chance that improved school leadership led to increased academic achievement among students.

In addition, Waters et al. (2003) said that the quality of a school’s leadership might have a substantial impact on student progress. Furthermore, a high correlation existed between an effective leadership style and student accomplishment. Similarly, leadership may have a negative impact on student success. According to (Broadbent, 2017) the strategic leadership style had a greater impact on school performance (an indication of maintaining student excellence) than the cultural leadership style in public schools as a whole and for male head teachers. In the instance of female head teachers, the cultural leadership style was much more influential than the strategic leadership style (toward shared objectives). The efficacy of the school led to the academic excellence of the students.

Furthermore, Ogbonnaya et al. (2020) did a research to determine the influence of transformational leadership style on students’ English language academic attainment. The research used a descriptive survey design. It was shown that transformational leadership had a good correlation with academic attainment among pupils.

Similarly, the results of a qualitative research done by Naz and Rashid (2021) indicate that instructional leadership may increase teacher motivation and improve student learning outcomes. Male and female instructors from both public and private secondary schools agreed that the instructional leaders fostered collaboration and fostered strong relationships between parents and school employees, hence sustaining kids’ academic performance.

Unanimity has not yet been reached over which leadership style produces the most successful organizational behavior. Different leadership styles are required for various circumstances, and each leader must understand when to use a certain style. Similarly, no one leadership style is optimal in every circumstance (Dahar et al., 2010; Allen et al., 2015).

The reviewed literature suggests that much study has been performed to report this phenomenon from various perspectives and in various circumstances. However, leftovers anonymous has yet to produce satisfactory results. In educational contexts, teaching leadership is defined as the capacity to plan, control, lead, organize, and manage human and material resources to achieve school goals and sustain academic excellence. Therefore, more clarity is required regarding education in Pakistan to cover the knowledge gap regarding how and which teaching leadership styles encourage and sustain academic success in secondary schools in Multan, Punjab, Pakistan.

## Materials and methods

### Study settings

In this study, the quantitative survey method was used (Ishtiaq, 2019), which involves multiple steps, including gathering and analyzing quantitative data to elaborate on prior understandings and hypotheses regarding a positive or negative relationship.

### Sampling

The current study includes a sample of 540 teachers in different positions like head of school, administration, and teachers of multi subjects. The sample included all teachers from secondary schools in Multan, Punjab, Pakistan. The region of Multan has 306 secondary schools from various small towns within the Board of Intermediate & Secondary Education (BISE) Multan. The participants were conveniently selected from 103 secondary schools.

### Instruments and data collection

A 33-item questionnaire was constructed to collect data about three types of (Strategic, Culture and Instructional leadership) contributing to academic excellence: strategic leadership with 10 items, cultural leadership with 8 items, instructional leadership with 9 items and sustaining academic excellence with 6 items. In order to assess teachers’ perspectives and based on the research topic, a 5-point Likert scale was used. From 1 (strongly disagree) to 5 (strongly agree). SPSS statistical software was used to analyze the collected data. For initiating the data collection, teachers were approached at their schools on consented days. The secondary school teachers participated voluntarily after receiving authorization and signing consent forms from the school principal. It was also making sure to them that they may withdraw their permission before the analysis. After analysis, the participant’s personal information and other relevant data were encrypted.

### Reliability and validity

Table 1 indicates the reliability statistics of leadership scales divided into three dimensions; Strategic Leadership, Culture Leadership, and Instructional Leadership. The reliability of the scales was conducted through Cronbach’s Alpha. Furthermore, the values were Strategic Leadership (*α* = 0.85), Culture Leadership (*α* = 0.82), Instructional Leadership (*α* = 0.78), and Sustaining Academic Excellence (*α* = 0.77) which was accurate, as suggested by the literature (Taber, 2018).

**Table 1:** Reliability analysis of scale dimensions.

Scale dimensions	Cronbach’s alpha
Strategic leadership	0.85
Culture leadership	0.82
Instructional leadership	0.78
Sustaining academic excellence	0.77

Before data analysis the Kolmogorov–Smirnov Test was employed to inspect the data’s normal distribution (Hanusz and Tarasińska, 2015). According to the outcome of the Kolmogorov–Smirnov test in Table 2, the data had a normal distribution and suitable for further analyses.

**Table 2:** The results of Kolmogorov–Smirnov test.

Instruments		Kolmogorov–Smirnov Z	Asymp. sig. (2-tailed)
Codes	Sub-variables		
SL	Strategic leadership	0.084	0.000
CL	Culture leadership	0.063	0.000
IL	Instructional leadership	0.075	0.000
Overall	Leadership styles	0.092	0.000
SEA	Sustain academic excellence	0.145	0.000

## Data analysis and findings

After conducting Kolmogorov–Smirnov Test, data was analyzed through different statistical analyses; Descriptive statistical analysis was conducted to elaborate the demographic variables. Means analysis was conducted to know the mean ranked and the role of various leadership styles. At the same time, bivariate correlation analysis was conducted to know the statistically significant relationship between diverse leadership styles and sustaining academic excellence.

### Demographic characteristic of participants

Table 3 shows participants’ demographic characteristics, divided into five variables: age, gender, academic qualifications, professional qualifications, and experience explained by frequency, percentage, mean and standard deviation. The age ranged were 26–35 (*f* = 309, % = 57.2), 36 above (*f* = 231, % = 42.8) with (*M* = 1.43 and SD = 0.495). Gender was male and female, in which male (*f* = 227, % = 42.0) female (*f* = 313, % = 58.0) with (*M* = 1.58 and SD = 0.494). Academic qualifications were categorized into; B.A/B.Sc. (*f* = 351, % = 65.0), M.A/M.Sc. (*f* = 170, % = 31.5), M.Phil. (*f* = 19, % = 3.5), with (*M* = 1.39 and SD = 0.555). Teachers professional diplomas were also classified into; B.Ed. (*f* = 405, % = 75.0), M.Ed. (*f* = 71, % = 13.1), and do not have (*f* = 64, % = 11.9) with (*M* = 1.37 and SD = 0.686). Finally, experience divided into 1–5 (*f* = 108, % = 20.0), 6–10 (*f* = 360, % = 67.7), and 11 above (*f* = 72, % = 13.3) with (*M* = 1.93 and SD = 0.574).

**Table 3:** Demographic characteristics of participants.

Variables		*F*	(%)	*M*	SD
Age	26–35	309	57.2	1.43	0.495
	36 Above	231	42.8		
Total		540	100.0		
Gender	Male	227	42.0	1.58	0.494
	Female	313	58.0		
Total		540	100.0		
Academic qualifications	B.A/B.Sc.	351	65.0	1.39	0.555
	M.A/M.Sc.	170	31.5		
	M.Phil.	19	3.5		
Total		540	100.0		
Professional qualifications	B.Ed.	405	75.0	1.37	0.686
	M.Ed.	71	13.1		
	Do not have	64	11.9		
Total		540	100.0		
Experience	1–5	108	20.0	1.93	0.574
	6–10	360	67.7		
	11 above	72	13.3		
Total		540	100.0		

*f*, frequency; %, percentage; *M*, mean; SD, standard deviation.

### Diversity in mean rank among leadership styles

Table 4 elaborates on the means analysis of each item regarding leadership style’s dimensions and overall. In which, Strategic Leadership explained with 10 items with overall (*M* = 36.34, SD = 7.55), Cultural Leadership explained with 8 items with overall (*M* = 29.21, SD = 6.45), and Instructional Leadership (*M* = 31.11, SD = 6.14). The means results revealed that Strategic Leadership scored the highest mean rank with value (*M* = 36.34) while Cultural Leadership scored the lowest mean rank (*M* = 29.21), which showed that the diversities in leadership styles play the most vital role in sustaining Academic Excellency at secondary school level in Punjab, Pakistan.

**Table 4:** Each item and overall mean scores and standard deviation of secondary schools teachers’ responses toward leadership styles.

Leaderships styles		*N*	*M* ± SD	Overall *M* ± SD	Rank
Strategic leadership	SL1	540	3.42 ± 1.139	36.34 ± 7.55	1
	SL2	540	3.24 ± 1.215		
	SL3	540	3.44 ± 1.270		
	SL4	540	3.38 ± 1.217		
	SL5	540	3.68 ± 1.219		
	SL6	540	3.62 ± 1.219		
	SL7	540	3.74 ± 1.143		
	SL8	540	4.09 ± 0.838		
	SL9	540	3.87 ± 1.124		
	SL10	540	3.91 ± 1.060		
Cultural leadership	CL1	540	3.66 ± 1.076	29.21 ± 6.45	3
	CL2	540	3.63 ± 1.088		
	CL3	540	3.53 ± 1.083		
	CL4	540	3.37 ± 1.137		
	CL5	540	3.23 ± 1.226		
	CL6	540	3.85 ± 1.240		
	CL7	540	3.91 ± 1.335		
	CL8	540	4.04 ± 1.346		
Instructional leadership	IL1	540	3.44 ± 1.224	31.11 ± 6.14	2
	IL2	540	3.26 ± 1.148		
	IL3	540	3.30 ± 1.182		
	IL4	540	3.74 ± 1.099		
	IL5	540	3.73 ± 1.058		
	IL6	540	3.49 ± 1.147		
	IL7	540	3.73 ± 1.087		
	IL8	540	3.23 ± 1.138		
	IL9	540	3.21 ± 1.108		

*N*, number of participants; *M*, mean; SD, standard deviation.

### Relationship of leadership styles and sustaining academic excellence

The results of Table 5 revealed that leadership styles in this study, such as; Strategic Leadership, Culture Leadership, and Instructional Leadership had a statistically significant positive relationship with sustaining academic excellence (*r* = 0.752). The results confirmed a significant and strong positive relationship between each independent variable (Strategic Leadership, Culture Leadership, and Instructional Leadership) and overall and the dependent variable (Sustaining Academic Excellence). Based on the findings, it has been discovered that when teachers increase the use of strategic, cultural, and instructional leadership styles, academic excellence may improve as well.

**Table 5:** Correlations among main variables.

	SL	CL	IL	SAE	Leadership styles
	1				
SL					
	540				
	0.535^**^	1			
CL	0.000				
	0.721^**^	0.620^**^	1		
IL	0.000	0.000			
	0.778^**^	0.598^**^	0.551^**^	1	
SAE	0.000	0.000	0.000		
	0.884^**^	0.819^**^	0.893^**^	0.752^**^	1
Leadership styles	0.000	0.000	0.000	0.000	

**Correlation is significant at the 0.01 level (2-tailed). SL, strategic leadership; CL, cultural leadership; IL, instructional leadership; SAE, sustaining academic excellence.

## Discussion

There has not been much research on how different leadership styles in secondary education teaching can help keep students doing well in school. Pakistan is a developing country there also needs to focus on the education sector, especially secondary education. Secondary education of students will generate a good and strong future in the coming days. The present study’s results showed that leadership styles play a vital role in sustaining academic excellence and that sustainable academic excellence has a statistically significant positive relationship with leadership styles.

However, research conducted by Hallinger (2011a) a forty-year-old institution, still stands strong today. Regarding leadership for learning, it is not about unveiling an innovation with a flourish or making a grand announcement. Instead, it refers to a continuous effort to improve learning conditions and develop coherence in daily values and actions across classrooms.

In contrast, Clarke-Habibi (2005) indicated the results of standardized instruments and school managers exhibit flexibility during their interaction. In addition, teachers are involved in the management of the school. Despite this, they are not sufficiently involved in strategic planning and the assessment of school performance. Administrative plans should employ a strategy that can be implemented within an organization and, in this way, demonstrate long-term planning while considering short-term concerns.

Moreover, instructional leadership has been the subject of numerous studies (Hallinger, 2011b), and has received worldwide attention, but there are still concerns regarding its meaning, practicality, and relevance. There is nearly a discussion among researchers regarding the lack of a comprehensive definition of IL (Shava et al., 2021). There is a lack of correlation between the implementation of the policy and the behaviors associated with it. The concept has also been criticized for focusing on transactional leadership, and superficial slogans are too prevalent (Hallinger and Bryant, 2013). However, educational research indicates that principals continue to spend most of their day administrating their schools despite recent attention to IL (Hallinger and Lee, 2014), In addition to school facilities management, school security, enforcement paperwork, and non-instructional programs (Irby, 2013), there is a question mark over the effectiveness of the implementation of IL (Williams 2019).

At the same time, Epling (2016) states that school culture plays a significant role in student achievement and contributes to an educational institution’s improvement. In addition to facilitating the program’s effectiveness. It also fosters communication and cooperation among all educational community members. Essentially, a school’s character sets it apart from other schools (González-Calvo et al., 2019). The perception of school units ultimately shapes their behavior (Richardson and Vandenberg, 2005). Rather than a static process, school culture possesses intrinsic features of a dynamic nature, shapes consciousness, and ultimately influences the development of two-way cooperative relationships among school community members.

The current study findings also supported earlier studies, such as; Day et al. (2016) concluded that self-governing leadership styles of institute managers were associated with positive effects on teachers’ performance. As summarize by Gerbi (2021) and Jay (2014), the leadership style of a school teacher is positively related to teacher performance that directly relates to students’ academic excellence, as indicate by Fatana (2021), that discovered a positive association among leadership style and instructors’ performance at work. The findings confirmed that teacher leadership styles are the most prevalent kind of leadership among instructors, followed by school leadership (Harris, 2002). Moreover, leadership styles significantly affect the teachers’ performance in the institution, which affects the teaching and learning process (Eyal and Roth, 2011). Lynch et al. (2016) synthesized 11 meta-analyses comprising almost 500 studies with over 1 million participants. It determined an overall effect of 0.36. As a result of leadership, a school’s ability to improve its performance can result in an increased leadership capacity in reciprocal relationships (Heck and Hallinger, 2010). Burns et al. (2017) compared the effects of different leadership activities; small to moderate benefits were reported for defining goals and expectations and fostering teacher learning and development. Ensure an ordered and positive teaching environment by planning, coordinating, and monitoring strategic resourcing. So, effective leadership for learning requires following school values, beliefs, and expectations. Borrowing from Mahatma Gandhi, “Be the change you want to you see in your school” (Robinson et al., 2008).

## Significance

### Practical

This study will be helpful for teachers because it will provide information to understand the role of teaching in various leadership styles. Identifying aspects may help the teachers to maximize sustaining academic excellence.

### Theoretical

The central theoretical significance of this study is its contribution to the literature on the importance of leadership styles and management that helps to review the role of teaching in sustaining academic excellence perspective.

## Conclusion

This study concludes by discussing the distinction between educational leadership styles and the role of teachers in determining and sustaining academic performance. If the leadership styles increase in teaching, then academic excellence will be sustained and improved. Leadership must be exemplified by a strong feeling of self-worth within the school and community, with no tendency toward self-regard or praise. In this scenario, the effective leader represents the school and community people. A leadership agenda that moralizes self-power that is necessary to balance the school’s excellence. There must be several things to establish desire, teacher autonomy, and external conscientious membership. This capability allows professional sagacity and originality, allowing for the cultivation of a productive learning environment. There is no opposition to the leadership domain’s criticism of researching teacher premises, and opposing beliefs to provide reflective evaluations and assessments.

## Limitations and future research directions

The current study also encounter limitations, the first, the sample is consisted of teachers only from one province. The further research can be done to include other educational stakeholders and provinces. While the second, only three leadership styles has chosen in this research. A comprehensive research can be conducted by exploring other leadership styles in other provinces of Pakistan.

## Data availability statement

The raw data supporting the conclusions of this article will be made available by the authors, without undue reservation.

## Ethics statement

The studies involving human participants were reviewed and approved by Zhejiang Normal University, Jinhua China. The patients/participants provided their written informed consent to participate in this study.

## Author contributions

SM: conceptualization, data curation, validation, and writing-original draft. HZ and LW: investigation, resources, and methodology. HZ, PZ, OK and TM: writing-review and editing. All authors contributed to the article and approved the submitted version.

## Conflict of interest

The authors declare that the research was conducted in the absence of any commercial or financial relationships that could be construed as a potential conflict of interest.

## Publisher’s note

All claims expressed in this article are solely those of the authors and do not necessarily represent those of their affiliated organizations, or those of the publisher, the editors and the reviewers. Any product that may be evaluated in this article, or claim that may be made by its manufacturer, is not guaranteed or endorsed by the publisher.
